# Health Risk Analysis of Elemental Components of an Industrially Emitted Respirable Particulate Matter in an Urban Area

**DOI:** 10.3390/ijerph18073653

**Published:** 2021-04-01

**Authors:** Oyewale Mayowa Morakinyo, Murembiwa Stanley Mukhola, Matlou Ingrid Mokgobu

**Affiliations:** 1 Department of Environmental Health, Faculty of Science, Tshwane University of Technology, Private Bag X680, Pretoria 0001, South Africa; mukholams@tut.ac.za (M.S.M.); mokgobumi@tut.ac.za (M.I.M.); 2 Department of Environmental Health Sciences, Faculty of Public Health, College of Medicine, University of Ibadan, Ibadan 200284, Nigeria

**Keywords:** heavy metals, PM_2.5_, hazard quotient, excess lifetime cancer risk, South Africa

## Abstract

Particulate matter of aerodynamic diameter of less than 2.5 µm (PM_2.5_) is a recognised carcinogen and a priority air pollutant owing to its respirable and toxic chemical components. There is a dearth of information in South Africa on cancer and non-cancer risks of exposure to heavy metal (HM) content of PM_2.5_. This study determined the seasonal concentration of HM in PM_2.5_ and the cancer and non-cancer risks of exposure to HM in PM_2.5_. Ambient PM_2.5_ was monitored and samples were collected during the winter and summer months in an industrialized area in South Africa. Concentration levels of nine HMs—As, Cu, Cd, Cr, Fe, Mn, Ni, Pb, and Zn—were determined in the PM_2.5_ samples using inductive coupled optical emission spectrophotometry. The non-cancer and cancer risks of each metal through the inhalation, ingestion and dermal routes were estimated using the Hazard Quotient and Excess Lifetime Cancer Risk (ELCR), respectively, among infants, children, and adults. Mean concentration of each HM-bound PM_2.5_ was higher in winter than in summer. The probability of the HM to induce non-cancer effects was higher during winter than in summer. The mean ELCR for HMs in PM_2.5_ (5.24 × 10^−2^) was higher than the acceptable limit of 10^−6^ to 10^−4^. The carcinogenic risk from As, Cd, Cr, Ni, and Pb were higher than the acceptable limit for all age groups. The risk levels for the carcinogenic HMs followed the order: Cr > As > Cd > Ni > Pb. The findings indicated that the concentrations of HM in PM_2.5_ demonstrated a season-dependent pattern and could trigger cancer and non-cancer health risks. The formulation of a regulatory standard for HM in South Africa and its enforcement will help in reducing human exposure to HM-bound PM_2.5_.

## 1. Introduction

South Africa represents one of the largest industrialised economies in the Southern Hemisphere with significant mining and metallurgical activities [1,2]. It is an arid country with high naturally occurring dust levels, coupled with industrial and vehicular pollution emissions [3]. Air quality in South Africa is characterised by a mixture of air pollution problems arising from urban-industrial activities and the domestic use of biofuels [4]. High Particulate matter (PM) pollution levels have been recorded in industrialised regions and urban areas in South Africa [5,6,7]. Additionally, many communities have constantly be exposed to experience high pollution levels from the use of unclean fuels, coal combustion and other mobile and stationary sources.

The PM_2.5_ denotes a toxic fraction of PM and is recognised as a major threat to human health due to its chemical toxicity and its ability to pass beyond the human larynx and ciliated airways [8,9]. It can penetrate the alveolar regions, blood circulation system [10] and getting into the extrapulmonary organs including the liver, spleen, heart, and brain [11].

Airborne PM is a heterogeneous mix of harmful chemical and biological substances including heavy metals, polycyclic aromatic hydrocarbons, fungi, bacteria and viruses. Heavy metals bounded to the particulate matter have been the focus in many environmental studies [12,13]. Heavy metals such as Cd, Cr, Cu, Mn, Ni, Pb, V, and Zn are typically present in PM [14] and have, therefore, been classified as an environmental priority pollutant by the United States Environmental Protection Agency [15]. Human exposure to heavy metal bounded PM is through inhalation, ingestion, and dermal absorption pathways [16,17].

Although HM in PM account for ~10% of its mass, it still denotes an environmentally key component due to its persistent bioavailability, long resident time in the environment and toxicity, even at low concentration thresholds [18]. Past epidemiological studies reported an association between exposure to PM-bound HMs and cardiovascular effects [19,20], myocardial toxicity-associated health risks, stroke [19], decreased functioning of the lung, pulmonary disease, bone defects, lung cancer [21,22], kidney damage and increased blood pressure [23,24]. Researchers assert that exposure to PM-bound metals can cause pulmonary toxicity [25], inflammatory responses, DNA damage and oxidative stress [26]. The International Agency for Research on Cancer classified As, Cd, Cr(VI) and Ni compound as human carcinogens [27] and inorganic Pb compounds as probable carcinogens [28]. Recently, Cr, Ni, Cu, Fe, V, and Zn have been reported to promote electron exchange [29] and aid the formation of reactive oxygen species in the lung [26].

In South Africa, the standard for PM_2.5_ was established in 2012 in terms of section 9(1) of the NEMA: AQA as 40 µg/m^3^ and 20 µg/m^3^ for a day and annual average, respectively [30]. Following the establishment of the standard, limited studies have determined the levels of PM_2.5_ in industrial areas. Additionally, no information currently exists on cancer and non-cancer risk assessment of exposure to HM bounded PM_2.5_ for specific age groups.

Having an understanding of the concentration of HM contained in PM_2.5_ is important for assessing the public health risk of exposure to PM_2.5_ as well as the potential non-cancer and cancer risks of exposure to HM in PM_2.5_. Additionally, this study will provide the evidence-based knowledge needed for the formulation of new environmental management plans for HM in South Africa. Therefore, this study aimed to determine the seasonal concentration of HM in PM_2.5_ and (2) the cancer and non-cancer health risks of exposure to HMbound PM_2.5_.

## 2. Materials and Methods

### 2.1. Study Area

The study was conducted in an industrial area in Pretoria West, located in the Gauteng province. It is situated to the north of Johannesburg and extends from Centurion in the south to Temba in the north, encompassing an area of 2200 km^2^ [31]. Sited in the industrial area are industrial facilities with approved air emission licences, power plants and metallurgical industries and facilities with small boilers [32]. The detailed information on the study area has been reported in our earlier published works [33,34,35].

### 2.2. Sampling Procedure for PM_2.5_

The Beta^PLUS^ Particle measurement system—model 602, which was part of an existing ambient air quality monitoring network sited at Pretoria West industrial area—was used for sampling of PM_2.5._ The Beta^PLUS^ Particle measurement system continuously measures PM_2.5_ mass concentration in the ambient air. Sampled PM_2.5_ was collected on a 47 mm glass fibre filter with a porosity of 2 µm by the Beta^PLUS^ Particle measurement system operating at a constant flow rate of 1 m^3^/h for 24 h. Filters were thereafter retrieved for heavy metal analysis. The full description of the sampling process and the gravimetric analysis of PM_2.5._ has been reported in our earlier publication [35].

### 2.3. Analysis of Heavy Metals in PM_2.5_

The analysis of HMs in PM_2.5_ was done following the procedure described by Hugelin et al. [36] To one-fourth of each of the 47 mm PM_2.5_ loaded quartz fiber filter portion in 25 mL flask was added 5 mL of 65% nitric acid (HNO_3_) and 1 mL of 30% hydrogen peroxide (H_2_O_2_) to extract the metals contained in PM_2.5_. The solution was then digested by placing the flask on a hot plate for about 24 h at a temperature of 100 °C. The temperature was later increased to 260 °C until white smoke appeared. A Whatman qualitative filter paper of diameter 47 mm was used for filtering the digested solution and to the effluent was added indium (internal standard) and 50 mL double distilled water. The solution was subsequently stored in refrigeration until instrumental analysis was done. Blank filters and duplicate samples were analysed following the same procedure earlier stated.

The concentrations of selected heavy metals in PM_2.5_ were determined using inductive coupled optical emission spectrophotometry (ICP-OES) (Perkin-Elmer Optima 2100 DV). Nine heavy metals: As, Cu, Cd, Cr, Fe, Mn, Ni, Pb, and Zn were determined in this study. Calibration of the instrument was done through the use of ICP-OES standard solution containing known amounts of the metals. For accuracy and precision of the test procedure, certified reference materials were used.

The values obtained for heavy metals were compared with the WHO air quality guidelines and the US EPA regulatory guideline (Table 1). It is important to note that the South African national ambient air quality standard (NAAQS) only has an exposure limit for Pb. Note that Mn and Zn recommended values were not seen in the literature.

### 2.4. Health Risk Assessment of HMs in PM_2.5_

The health risk assessment (HHRA) model adopted for the estimation of the non-cancer and cancer risks from exposure to HMs (As, Cu, Cd, Cr, Fe, Mn, Ni, Pb, and Zn) in PM_2.5_ was based on the US EPA human health evaluation method [38]. Human exposure to a heavy metal component of PM_2.5_ can occur through ingestion, inhalation and dermal routes [39,40]. Human exposure was explained in terms of average daily dose and was computed for each metal and each exposure routes as follows:

*ADD_inh_* = *C* ∗ *InhR* ∗ *EF* ∗ *ED* (1)

*BW* ∗ *AT*

*ADD_ing_* = *C* ∗ *IngR* ∗ *EF* ∗ *ED* (2)

*BW* ∗ *AT*

*ADD_derm_* = *C* ∗ *SA* ∗ *AF* ∗ *ABS* ∗ *EF* ∗ *ED* (3)

*BW* ∗ *AT*

where *ADD_inh_*, *ADD_ing_*, *ADD_derm_* is the average daily dose of each metal in PM_2.5_ through the inhalation, ingestion and dermal routes; *C* is the amount of PM_2.5_ in ambient air (μg/m^3^); *ED* is the exposure duration (days), *BW* is the body weight of the exposed group (kg); *AT* is the averaging time (days); *IngR* is the ingestion rate (mg/day), *InhR* is the inhalation rate (m^3^/day); *SA* is the surface area of the skin exposed to pollutants (cm^2^), *AF* is the skin adherence factor (mg/cm^2^/day), *ABS* is the dermal absorption factor [41,42]; *EF* is the exposure frequency (days/year). An *EF* of 350 days per year was used to calculate the lifetime exposure of human receptors (both child and adult) with the assumption that all population in Pretoria West spends a maximum of 14 days per year away from the study area [43]. The values of these parameters are stated in Table 2.

### 2.5. Estimation of Non-Carcinogenic Risks of Heavy Metals in PM_2.5_

The non-carcinogenic risk of each metal through the inhalation, ingestion and dermal routes was estimated using the Hazard Quotient (*HQ*). This was achieved by dividing the *ADD* from each exposure route by a definite reference dose (*RfD*). The *HQ* is defined as follows:

*HQ* = *ADD* (4)

*RfD*

For the inhalation route,

*HQ* = *ADD* (5)

*RfCi* × 1000 µg mg^−1^

where *RfD* (reference dose, mg kg^−1^day^−1^) is the estimated maximum allowable risk to humans from daily exposure to a known pollutant [40]; *RfCi* is inhalation reference concentrations (mg m^−3^). An *HQ* of less than 1 signifies that no adverse health risk will occur while and *HQ* of more than 1 signifies that potential non-cancer effects would occur [48]. The *RfD* values used in estimating the *HQ* are presented in Table 3.

To determine the possible non-cancer effects that could arise from exposure to the synergistic effects of several metals, the sum of *HQ* values of all the metals were computed and expressed as a hazard index (*HI*):

(6) $$HI=\sum_{i=1}^{n}HQ1+HQ2+\ldots +HQi$$

where *HQi* denotes the *HQ* for the *i*th element. *HI* < 1 stands for no significant risk of non-carcinogenic effects while *HI* > 1 shows the likely occurrence of non-carcinogenic effects [49].

The non-carcinogenic risks through the total exposure route (*HIt*) were calculated as the sum of all HI for the inhalation, ingestion and dermal routes. It was expressed as follows:

(7) $$\sum_{1}^{i}HI$$

When *HIt* is less than 1, protracted health risks are unlikely to occur, although non-carcinogenic risks are likely to occur when *HIt* > 1 [50].

### 2.6. Estimation of Carcinogenic Risks of Heavy Metals in PM_2.5_

The Excess Lifetime Cancer Risk (*ELCR*—likelihood of developing cancer over the lifespan of an individual as a consequence of exposure to a carcinogenic metal) of exposure to As, Cd, Cr, Ni, and Pb was computed as follows:

*ELCR* = *EC* × *IUR* (8)

where *EC* is the exposure concentration via the inhalation route; *IUR* (mg m^−3^)^−1^ is the inhalation unit risk of HMs in PM_2.5_. The *RfCi* and *IUR* values are shown in Table 4.

The exposure concentration was estimated as:

*EC* = *C* ∗ *InhR* ∗ *EF* ∗ *ED* (9)

ATn

where ATn is the average time for carcinogens (70-year × 365 days/year × 24 h/day) [38,39].

The *IUR* of carcinogenic risk through the ingestion and dermal routes were not provided in the literature, so only the carcinogenic risk of HMs in PM_2.5_ through the inhalation pathway was computed. The allowable or tolerable ELCR for the regulatory purpose is 1 × 10^−6^–1 × 10^−4.3^.

## 3. Results and Discussion

### 3.1. PM_2.5_-Bound Heavy Metal Concentration

Higher mean concentrations of metals in PM_2.5_ were found in winter than in summer (Table 5). The winter/summer concentration ratio of greater than 1 was observed for all metals: As (1.4), Cu (1.4), Cd (1.2), Cr (1.1), Fe (1.2), Mn (1.1), Ni (1.7), Pb (1.4) and Zn (1.2). In terms of non-carcinogenic metals, the pattern of Fe > Cu > Zn > Mn was observed whereas, the pattern was Pb > Ni > As > Cd, > Cr for carcinogenic metals in winter and summer months, respectively. Similar studies carried out in other countries such as China [51], India [37], Italy [52], reported a higher mean concentrations of metals in winter than in summer.

A plausible explanation for increased metal concentration in PM during winter was attributed to stable weather conditions occasioned by less precipitation, stagnation of air movement, low wind speed and turbulence, thermal and strong inversion, and high relative humidity that are predominant during winter [53].

Furthermore, the metal-bound PM_2.5_ measured in this study was compared with national and international regulatory guidelines shown in Table 1. The mean values of As, Cd, Cr, Mn, Ni, Pb, and Zn were higher than the recommended exposure safe limit. Higher concentrations of HMs in PM exceeding recommended limits have been reported [54]. The proximity of the monitoring station to the emission sources like the power plants and metallurgical industries in the study site may play a significant role in the higher concentration of metals that was recorded. Arsenic, Pb, Cd, Cr and Ni in PM had been reported to be markers of industrial emissions majorly from coal-fired power plants, metallurgical industries, and oil refineries [55].

### 3.2. Average Daily Exposure Dose

The average daily exposure dose (ADD), which is the dose rate of heavy metals (HMs) in PM_2.5_ via the ingestion, dermal and inhalation exposure pathways, is expressed as a daily dose on a per-unit-body-weight basis is presented in Table 6. The ADD of HMs in PM_2.5_ through the three exposure pathways for the different age groups followed the pattern Cu > Fe > Zn > Mn > Pb > Ni > As > Cd > Cr.

The ADD of the HMs also differs for the different exposure groups. For example, the ADDs of Fe, Mn, and Zn were greater for infants than for adults while the ADDs of As, Cr, Ni, and Pb were highest for adults than for infants.

### 3.3. Non-Carcinogenic Risks of HMs in PM_2.5_

The non-carcinogenic risks of HMs in PM_2.5_ via dermal contact, ingestion and inhalation exposure pathways are shown in Table 7. Findings from this study showed that the probability of exposure to HMs to induce non-cancer effects was higher during winter than in summer. Cadmium, Mn, and Ni may induce non-carcinogenic effects (*HQ* > 1) through the inhalation routes for all age groups while Mn is the only HM in PM_2.5_ that may cause non-carcinogenic health effects through dermal contact. Arsenic possessed significant non-cancer risks for all age groups through the inhalation and ingestion routes. None of the HMs possessed the ability to induce non-cancer through the dermal contact, ingestion and inhalation exposure pathways.

Epidemiological studies conducted in the past have reported an association between exposure to elevated levels of some metals such as Ni, Cd, Cu, and As in PM_2.5_ and markers of cardiovascular disease [19,56]. The association between exposure to Cd and the occurrence of hypertension resulting in atherosclerosis and myocardial infarction was established in an epidemiological study. In toxicological studies, the incidence of hyperglycemia, insulin resistance and glycemic deregulation from exposure to Ni have been reported [57,58].

In this study, there was no consistent pattern for the non-cancer effects across the age groups. For instance, through the inhalation route, As, Cr, and Ni will likely induce the highest non-cancer effects in adults; Cu and Mn will cause greater health outcomes in infants while it is in children for Cd. Children were previously identified to be more prone to the adverse effects of pollutants than adults [49,59]. The reason being that children breathe in more air per their unit body weight, and their not fully formed immune system cannot handle environmental pollutants amidst other physiological [60,61]. However, a possible explanation for the higher susceptibility of adults to non-carcinogenic effects of HMs in PM_2.5_ was that adults engaged more in physically demanding activities that required a higher rate of inhalation than children.

Furthermore, the hazard index (*HI*) of all the HMs in this study, computed as the sum of all *HQ*s for individual metals for each pathway [49], is presented in Table 6. It was observed that the cumulative non-carcinogenic effects of the all the HMs through the different exposure routes exceeded the safe limit of 1. This implied that the probability that non-carcinogenic effects will occur from exposure to the synergy of HMs in PM_2.5_ was higher compared to the risk from exposure to individual metals. This also suggested that the metals would have a cumulative non-cancer effect on all age groups. Seasonal variation in *HI* of metals for the different age groups was also observed with the *HI* value been greater than 1 during winter months. Incidence of the *HI* for multi-elemental exposure exceeding safe the limit has also been reported [42].

### 3.4. Carcinogenic Risks of PM_2.5_-Bound HMs

Findings from this study showed that the total average value of the ELCR for HMs in PM_2.5_ was 5.24 × 10^−2^, which is higher than the acceptable limit of 10^−6^ to 10^−4^ (Table 8). The carcinogenic risk from As, Cd, Cr, Ni, and Pb were higher than the acceptable limit for all age groups except for Ni and Pb that will not likely induce any cancerous effects in infants. The risk levels for the carcinogenic HMs followed the order: Cr > As > Cd > Ni > Pb. In addition, these HMs posed the greatest cancer risk to adults, then to children and lastly to infants. Similar epidemiological studies have reported higher cancer risks in adults than in infants [39,50,62]. Hu and his colleagues reported that adults have higher exposure time to carcinogenic HMs, hence the greater the dose of HMs accumulated in the body [39]. This study also found that cancer risk increased during winter (2.75 × 10^−2^) compared to summer (2.50 × 10^−2^). Raaschou-Nielsen and colleagues have earlier reported an association between the incidence of lung cancer and exposure to heavy metal contents of PM_2.5_ in a cohort study conducted in fourteen European countries [25].

#### Limitation of Study

One of the limitations of this study was the assumption that the levels of PM_2.5_ recorded by the fixed monitoring station are representative of the total concentration of PM_2.5_ to which the residents within the study area are exposed. However, PM concentration obtained from a fixed site may not be a true measurement of personal PM exposure due to individual activities and other factors. Additionally, certain uncertainties are associated with the use of the health risk assessment framework. However, the human health risk assessment framework has been useful in quantitative studies for estimating health risks and for making informed decisions in regarding risk abatement.

## 4. Conclusions

In this study, the seasonal concentrations of PM_2.5_-bound HM and their cancer and non-cancer risks in the Pretoria West industrial area were determined. The findings indicated that the concentrations of HM in PM_2.5_ demonstrated a season-dependent pattern.

The average concentration of As, Cd, Cr, Mn, Ni, Pb, and Zn in PM_2.5_ exceeded the recommended safe limit for humans. For all age groups, there is the likelihood that Fe, Pb, and Zn will induce non-cancer risk through the inhalation, ingestion and dermal pathways. Exposure to the cumulative effects of all HMs in PM_2.5_ through both the individual and the total pathways will result in non-carcinogenic health effects. Arsenic, Cd, Cr, Ni, and Pb have higher cancer risk than safe limits with them posing the greatest cancer risk to adults and least to infants.

The results provided evidence that the levels of HM in the study area may be a threat to human health. It also gave additional insights into the pollution issues in the study area and it is a pointer to the need for more rigorous strategies for controlling emissions. These findings would be useful for policymakers and relevant stakeholders in coming up with measures that will mitigate trace metal concentrations in PM. New air quality guidelines for HM should be established in South Africa. Besides Pb, there are no existing regulatory standards for HM in South Africa. Additionally, industrial processes should be monitored by the government by enforcing the installation of newer technologies that produce cleaner emissions. This study recommends future studies focusing on determining the effects of other components of PM_2.5_ from varied sources on measures of health outcomes in different urban areas in South Africa.

## Figures and Tables

**Table 1:** Permissible limits of concentrations of metals in ambient air.

Metal	Winter (µg/m^3^)	Summer (µg/m^3^)	Limit Value (µg/m^3^)
As	0.035	0.07	0.006
Cd	0.026	0.022	0.0002
Cr	0.354	0.309	0.012
Cu	0.2	0.2	100
Fe	4.3	3.4	10,000
Ni	0.067	0.061	0.00024
Pb	0.5	0.5	0.5

NAAQS: National Ambient Air Quality Standards; USEPA: United States Environmental Protection Agency; NIOSH: National Institute for Occupational Safety and Health. Source: Agarwal et al. [37].

**Table 2:** Recommended values in equations of the daily exposure dose of PM_2.5._

Parameter	Definition	Value for Age Categories				Reference
		Infant (0–1 yr)	Child (2–5 yrs)	Child (6–12 yrs)	Adult (19–75 yrs)	
*C*	Mean concentration of PM_2.5_ in ambient air (μg/m^3^)					
*IngR*	Ingestion rate (mg/day)	60	60	60	30	US EPA [44]
*EF*	Exposure frequency (days/year)	350	350	350	350	Morakinyo et al. [7], US EPA [45]
*ED*	Exposure duration (years)	1	6	12	30	Matooane and Diab [46] US EPA [45]
*ET*	Exposure time (h)	1	8	6	3	Matooane and Diab [46] US EPA [45]
*AT*	Averaging time (days); *AT* = *ED* × 365 days	365	2190	4380	10,950	Matooane and Diab [46] US EPA [45]
*BW*	Body weight (kg)	11.3	22.6	45.3	71.8	Matooane and Diab [45]
*SA*	Skin surface area (cm^2^)	2800	2800	2800	5700	US EPA [47]
*AF*	Adherence factor of soil to skin (mg/cm^2^/event)	0.2	0.2	0.2	0.07	US EPA [47]
*ABS*	Dermal absorption fraction	0.001	0.001	0.001	0.001	US EPA [47]
*InhR*	Inhalation rate (m^3^/day)	9.2	16.74	21.02	21.4	US EPA [45]

**Table 3:** Recommended values of Reference Doses.

	As	Cu	Cd	Cr	Fe	Mn	Ni	Pb	Zn
RfD-ADDing	3.00 × 10^−04^	4.00 × 10^−02^	1.00 × 10^−03^	3.00 × 10^−03^	-	4.60 × 10^−02^	2.00 × 10^−02^	3.50 × 10^−03^	3.00 × 10^−01^
RfD-ADDderm	1.23 × 10^−04^	4.02 × 10^−02^	1.00 × 10^−05^	2.86 × 10^−05^	-	1.43 × 10^−05^	2.06 × 10^−02^	5.25 × 10^−04^	6.00 × 10^−02^
RfDi-ADDinh	1.50 × 10^−05^	1.20 × 10^−02^	1.00 × 10^−05^	1.00 × 10^−04^	7.00 × 10^−01^	5.00 × 10^−05^	1.40 × 10^−05^	3.50 × 10^−03^	3.00 × 10^−01^

Source: Izhar et al. [40] Note: RfD-ADDing: Ingestion reference dose (mg/kg/day), RfD-ADDderm: Dermal contact reference dose (mg/kg/day), RfDi-ADDinh: Inhalation reference dose (mg/kg/day).

**Table 4:** Recommended values of *RfC* and *IUR*.

	As (Inorganic)	Cd (Diet/Water)	Cr (VI)	Mn (Diet)	Ni	Pb
*RfC* (mg/m^3^)	1.50 × 10^−05^	1.00 × 10^−05^	1.00 × 10^−04^	5.00 × 10^−05^	1.40 × 10^−05^	-
*IUR* (mg/m^3^)^−1^	4.30 × 10^−03^	1.80 × 10^−03^	8.40 × 10^−02^	-	2.40 × 10^−04^	1.20 × 10^−05^

The *RfC* and *IUR* were obtained from the US EPA website—http://www.epa.gov/region9/superfund/prg/index.html (accessed on 9 April 2016) [38] and the study of Li et al. [51].

**Table 5:** Seasonal variation of ions and metals in PM_2.5._

Heavy Metal	Winter		Summer	
	Mean (μg/m^3^)	SD	Mean (μg/m^3^)	SD
As	4.72	3.97	3.32	2.68
Cu	7.31	5.44	5.23	3.50
Cd	2.81	2.79	2.28	2.23
Cr	2.39	2.10	2.18	1.23
Fe	7.10	5.34	5.71	2.63
Mn	1.66	1.01	1.58	1.05
Ni	6.29	3.67	5.40	3.32
Pb	8.48	6.21	5.83	4.24
Zn	3.23	1.70	2.76	1.78

**Table 6:** Average daily dose of HMs in PM_2.5_ via inhalation, ingestion, and dermal routes.

Metal	Season	ADD Inhalation				ADD Ingestion				ADD Dermal			
		Infant	Child	Child	Adult	Infant	Child	Child	Adult	Infant	Child	Child	Adult
As	Winter Summer	5.26 × 10^−02^ 3.70 × 10^−02^	2.87 × 10^−01^ 2.02 × 10^−01^	3.60 × 10^−01^ 2.53 × 10^−01^	5.78 × 10^−01^ 4.07 × 10^−01^	3.43 × 10^−01^ 2.41 × 10^−01^	1.03 × 10^+00^ 7.25 × 10^−01^	1.03 × 10^+00^ 7.22 × 10^−01^	8.10 × 10^−01^ 5.70 × 10^−01^	3.20 × 10^−03^ 2.25 × 10^−03^	9.61 × 10^−03^ 6.76 × 10^−03^	9.59 × 10^−03^ 6.75 × 10^−03^	1.08 × 10^−02^ 7.58 × 10^−03^
Cu	Winter Summer	5.71 × 10^+00^ 4.25 × 10^+00^	5.91 × 10^+00^ 3.86 × 10^+00^	3.25 × 10^+00^ 2.42 × 10^+00^	2.09 × 10^+00^ 1.53 × 10^+00^	3.72 × 10^+01^ 2.66 × 10^+01^	1.86 × 10^+01^ 1.33 × 10^+01^	9.28 × 10^+01^ 6.64 × 10^+01^	2.93 × 10^+01^ 2.10 × 10^+01^	3.47 × 10^−01^ 2.49 × 10^−01^	1.74 × 10^−01^ 1.24 × 10^−01^	8.67 × 10^−02^ 6.20 × 10^−02^	3.90 × 10^−02^ 2.79 × 10^−02^
Cd	Winter Summer	3.13 × 10^−02^ 2.54 × 10^−02^	1.71 × 10^−01^ 1.39 × 10^−01^	2.14 × 10^−01^ 1.74 × 10^−01^	3.44 × 10^−01^ 2.79 × 10^−01^	2.04 × 10^−01^ 1.67 × 10^−01^	6.13 × 10^−01^ 5.00 × 10^−01^	6.12 × 10^−01^ 5.00 × 10^−01^	4.80 × 10^−01^ 3.91 × 10^−01^	1.91 × 10^−03^ 1.55 × 10^−03^	5.72 × 10^−03^ 4.64 × 10^−03^	5.71 × 10^−03^ 4.63 × 10^−03^	6.42 × 10^−03^ 5.21 × 10^−03^
Cr	Winter Summer	2.67 × 10^−02^ 2.43 × 10^−02^	1.46 × 10^−01^ 1.33 × 10^−01^	1.82 × 10^−01^ 1.66 × 10^−01^	2.93 × 10^−01^ 2.67 × 10^−01^	1.74 × 10^−01^ 1.59 × 10^−01^	5.22 × 10^−01^ 4.76 × 10^−01^	5.20 × 10^−01^ 4.75 × 10^−01^	4.10 × 10^−01^ 3.74 × 10^−01^	1.62 × 10^−03^ 1.48 × 10^−03^	4.87 × 10^−03^ 4.44 × 10^−03^	4.86 × 10^−03^ 4.43 × 10^−03^	5.46 × 10^−03^ 4.98 × 10^−03^
Fe	Winter Summer	5.54 × 10^+00^ 4.46 × 10^+00^	5.04 × 10^+00^ 4.06 × 10^+00^	3.16 × 10^+00^ 2.54 × 10^+00^	2.03 × 10^+00^ 1.63 × 10^+00^	3.61 × 10^+01^ 2.91 × 10^+01^	1.81 × 10^+01^ 1.45 × 10^+01^	9.02 × 10^+00^ 7.25 × 10^+00^	2.84 × 10^+00^ 2.29 × 10^+00^	3.37 × 10^−01^ 2.71 × 10^−01^	1.69 × 10^−01^ 1.36 × 10^−01^	8.42 × 10^−02^ 6.77 × 10^−02^	3.78 × 10^−02^ 3.04 × 10^−02^
Mn	Winter Summer	1.30 × 10^+00^ 1.23 × 10^+00^	1.18 × 10^+00^ 1.12 × 10^+00^	7.39 × 10^−01^ 7.03 × 10^−01^	4.74 × 10^−01^ 4.52 × 10^−01^	8.45 × 10^+00^ 8.05 × 10^+01^	4.23 × 10^+00^ 4.02 × 10^+01^	2.11 × 10^+00^ 2.01 × 10^+00^	6.65 × 10^−01^ 6.33 × 10^−01^	7.89 × 10^−02^ 7.51 × 10^−02^	3.94 × 10^−02^ 3.73 × 10^−02^	1.97 × 10^−02^ 1.87 × 10^−02^	8.85 × 10^−03^ 8.42 × 10^−03^
Ni	Winter Summer	7.02 × 10^−02^ 6.02 × 10^−02^	3.83 × 10^−01^ 3.29 × 10^−01^	4.80 × 10^−01^ 4.11 × 10^−01^	7.70 × 10^−01^ 6.61 × 10^−01^	4.58 × 10^−01^ 3.93 × 10^−01^	1.37 × 10^+00^ 1.18 × 10^+00^	1.37 × 10^+00^ 1.18 × 10^+00^	1.08 × 10^+00^ 9.27 × 10^−01^	4.27 × 10^−03^ 3.67 × 10^−03^	1.28 × 10^−02^ 1.10 × 10^−02^	1.28 × 10^−02^ 1.10 × 10^−02^	1.44 × 10^−02^ 1.23 × 10^−02^
Pb	Winter Summer	9.46 × 10^−02^ 6.50 × 10^−02^	5.16 × 10^−01^ 3.55 × 10^−01^	6.47 × 10^−01^ 4.50 × 10^−01^	1.04 × 10^+00^ 7.14 × 10^−01^	6.17 × 10^−01^ 4.24 × 10^−01^	1.85 × 10^+00^ 1.27 × 10^+00^	1.85 × 10^+00^ 1.27 × 10^+00^	1.46 × 10^+00^ 1.00 × 10^+00^	5.76 × 10^−03^ 3.96 × 10^−03^	1.73 × 10^−02^ 1.19 × 10^−02^	1.72 × 10^−02^ 1.18 × 10^−02^	1.94 × 10^−02^ 1.33 × 10^−02^
Zn	Winter Summer	2.52 × 10^+00^ 2.15 × 10^+00^	2.29 × 10^+00^ 1.96 × 10^+00^	1.44 × 10^+00^ 1.23 × 10^+00^	9.23 × 10^−01^ 7.89 × 10^−01^	1.65 × 10^+01^ 1.41 × 10^+01^	8.22 × 10^+00^ 7.03 × 10^+00^	4.10 × 10^+00^ 3.51 × 10^+00^	1.29 × 10^+00^ 1.11 × 10^+00^	1.53 × 10^−01^ 1.31 × 10^−01^	7.67 × 10^−02^ 6.56 × 10^−02^	3.83 × 10^−02^ 3.27 × 10^−02^	1.72 × 10^−02^ 1.47 × 10^−02^

**Table 7:** Summary of non-carcinogenic risks of HMs in PM_2.5_ via dermal contact, ingestion and inhalation exposure pathways.

Metal	Season	*HQ* Inhalation				*HQ* Ingestion				*HQ* Dermal			
		Infant	Child	Child	Adult	Infant	Child	Child	Adult	Infant	Child	Child	Adult
As	Winter Summer	**3.51 × 10^+00^** **2.47 × 10^+00^**	**1.91 × 10^+01^** **1.35 × 10^+01^**	**2.40 × 10^+01^** **1.69 × 10^+01^**	**3.85 × 10^+01^** **2.71 × 10^+01^**	**1.14 × 10^+00^** 8.03 × 10^−01^	**3.43 × 10^+00^** **2.42 × 10^+00^**	**3.43 × 10^+00^** **2.41 × 10^+00^**	**2.70 × 10^+00^** **1.90 × 10^+00^**	2.60 × 10^−02^ 1.83 × 10^−02^	7.81 × 10^−02^ 5.50 × 10^−02^	7.80 × 10^−02^ 5.49 × 10^−02^	8.78 × 10^−02^ 6.16 × 10^−02^
Cu	Winter Summer	**4.08 × 10^+02^** **3.04 × 10^+02^**	**4.22 × 10^+02^** **2.76 × 10^+02^**	**2.32 × 10^+02^** **1.73 × 10^+02^**	**1.49 × 10^+02^** **1.09 × 10^+02^**	9.30 × 10^−01^ 6.65 × 10^−01^	4.65 × 10^−01^ 3.33 × 10^−01^	**2.32 × 10^+00^** **1.66 × 10^+00^**	7.33 × 10^−01^ 5.25 × 10^−01^	8.26 × 10^−03^ 5.93 × 10^−03^	4.14 × 10^−03^ 2.95 × 10^−03^	2.06 × 10^−03^ 1.48 × 10^−03^	9.29 × 10^−04^ 6.64 × 10^−04^
Cd	Winter Summer	**3.13 × 10^+00^** **2.54 × 10^+00^**	**1.71 × 10^+02^** **1.39 × 10^+01^**	**2.41 × 10^+01^** **1.74 × 10^+01^**	**3.44 × 10^+01^** **2.79 × 10^+01^**	2.04 × 10^−01^ 1.67 × 10^−01^	6.13 × 10^−01^ 5.00 × 10^−01^	6.12 × 10^−01^ 5.00 × 10^−01^	4.80 × 10^−01^ 3.91 × 10^−01^	1.91 × 10^−01^ 1.55 × 10^−01^	5.72 × 10^−01^ 4.64 × 10^−01^	5.71 × 10^−01^ 4.63 × 10^−01^	6.42 × 10^−01^ 5.21 × 10^−01^
Cr	Winter Summer	2.67 × 10^−01^ 2.43 × 10^−01^	**1.46 × 10^+00^** **1.33 × 10^+00^**	**1.82 × 10^+00^** **1.66 × 10^+00^**	**2.93 × 10^+00^** **2.67 × 10^+00^**	5.80 × 10^−02^ 5.30 × 10^−02^	1.74 × 10^−01^ 1.59 × 10^−01^	1.73 × 10^−01^ 1.58 × 10^−01^	1.37 × 10^−01^ 1.25 × 10^−01^	5.66 × 10^−02^ 5.18 × 10^−02^	1.70 × 10^−01^ 1.55 × 10^−01^	1.70 × 10^−01^ 1.55 × 10^−01^	1.91 × 10^−01^ 1.74 × 10^−01^
Fe	Winter Summer	7.91 × 10^−03^ 6.37 × 10^−03^	7.20 × 10^−03^ 5.80 × 10^−03^	4.51 × 10^−03^ 3.63 × 10^−03^	2.90 × 10^−03^ 2.33 × 10^−03^	5.16 × 10^−02^ 4.16 × 10^−02^	2.59 × 10^−02^ 2.07 × 10^−02^	1.29 × 10^−02^ 1.04 × 10^−02^	4.06 × 10^−03^ 3.27 × 10^−03^	4.81 × 10^−04^ 3.87 × 10^−04^	2.41 × 10^−04^ 1.94 × 10^−04^	1.20 × 10^−04^ 9.67 × 10^−05^	5.40 × 10^−05^ 4.34 × 10^−05^
Mn	Winter Summer	**2.60 × 10^+01^** **2.46 × 10^+01^**	**2.36 × 10^+01^** **2.24 × 10^+01^**	**1.48 × 10^+01^** **1.41 × 10^+01^**	**9.48 × 10^+00^** **9.04 × 10^+00^**	1.84 × 10^−01^ 1.75 × 10^−01^	9.20 × 10^−02^ 8.74 × 10^−01^	4.59 × 10^−02^ 4.37 × 10^−02^	1.45 × 10^−02^ 1.38 × 10^−02^	**5.52 × 10^+00^** **5.25 × 10^+00^**	**2.76 × 10^+00^** **2.61 × 10^+00^**	**1.38 × 10^+00^** **1.31 × 10^+00^**	6.19 × 10^−01^ 5.89 × 10^−01^
Ni	Winter Summer	**5.01 × 10^+00^** **4.30 × 10^+00^**	**2.74 × 10^+01^** **2.35 × 10^+01^**	**3.43 × 10^+01^** **2.94 × 10^+01^**	**5.50 × 10^+01^** **4.72 × 10^+01^**	2.29 × 10^−02^ 1.97 × 10^−02^	6.85 × 10^−02^ 5.90 × 10^−02^	6.85 × 10^−02^ 5.90 × 10^−02^	5.40 × 10^−02^ 4.64 × 10^−02^	2.07 × 10^−04^ 1.78 × 10^−04^	6.21 × 10^−04^ 5.34 × 10^−04^	6.21 × 10^−04^ 5.34 × 10^−04^	6.99 × 10^−04^ 5.97 × 10^−04^
Pb	Winter Summer	2.69 × 10^−02^ 1.85 × 10^−02^	1.47 × 10^−01^ 1.01 × 10^−01^	1.84 × 10^−01^ 1.28 × 10^−01^	2.95 × 10^−01^ 2.03 × 10^−01^	1.76 × 10^−01^ 1.21 × 10^−01^	5.29 × 10^−01^ 3.63 × 10^−01^	5.29 × 10^−01^ 3.63 × 10^−01^	4.17 × 10^−01^ 2.86 × 10^−01^	1.10 × 10^−02^ 7.54 × 10^−03^	3.30 × 10^−02^ 2.27 × 10^−02^	3.28 × 10^−02^ 2.25 × 10^−02^	3.70 × 10^−02^ 2.53 × 10^−02^
Zn	Winter Summer	8.37 × 10^−03^ 7.14 × 10^−03^	7.61 × 10^−03^ 6.51 × 10^−03^	4.78 × 10^−03^ 4.09 × 10^−03^	3.07 × 10^−03^ 2.62 × 10^−03^	5.50 × 10^−02^ 4.70 × 10^−02^	2.74 × 10^−02^ 2.34 × 10^−02^	1.37 × 10^−02^ 1.17 × 10^−02^	4.30 × 10^−03^ 3.70 × 10^−03^	2.55 × 10^−03^ 2.18 × 10^−03^	1.28 × 10^−03^ 1.09 × 10^−03^	6.38 × 10^−04^ 5.45 × 10^−04^	2.87 × 10^−04^ 2.45 × 10^−04^
HI	Winter Summer	**4.46 × 10^+02^** **3.38 × 10^+02^**	**6.65 × 10^+02^** **3.51 × 10^+02^**	**3.31 × 10^+02^** **2.53 × 10^+02^**	**2.90 × 10^+02^** **1.81 × 10^+02^**	**2.82 × 10^+00^** **3.67 × 10^+00^**	**5.42 × 10^+00^** **4.75 × 10^+00^**	**7.21 × 10^+00^** **5.22 × 10^+00^**	**4.54 × 10^+00^** **3.29 × 10^+00^**	**5.82 × 10^+00^** **5.49 × 10^+00^**	**7.76 × 10^+00^** **3.31 × 10^+00^**	**2.24 × 10^+00^** **2.01 × 10^+00^**	**1.58 × 10^+00^** **1.37 × 10^+00^**
Hit			**Infant**		**Child**		**Toddler**			**Adult**			
	Winter Summer		4.55 × 10^+02^ 3.47 × 10^+02^		6.78 × 10^+02^ 3.59 × 10^+02^		3.41 × 10^+02^ 2.60 × 10^+02^			2.96 × 10^+02^ 1.86 × 10^+02^			

The values in bold depict that the levels of the heavy metals through the different exposure pathways were exceeded; *HI* (∑*HQ*): Represents the sum *HQ* value of 9 heavy metals in winter and summer, *HIt* (∑*HI*): Represents the sum *HI* value of three exposure pathways.

**Table 8:** Carcinogenic risks via inhalation exposure to heavy metals in PM_2.5._

Metal	Season	*EC* Inhalation				*IUR* (µg/m^3^)^−1^	ECLR			
		Infant	Child	Child	Adult		Infant	Child	Toddler	Adult
As	Winter Summer	2.69 × 10^−03^ 1.90 × 10^−03^	1.29 × 10^−01^ 9.10 × 10^−02^	1.94 × 10^−01^ 1.36 × 10^−01^	2.42 × 10^−01^ 1.71 × 10^−01^	4.30 × 10^−03^	1.16 × 10^−05^ 8.17 × 10^−06^	5.55 × 10^−04^ 3.91 × 10^−04^	8.34 × 10^−04^ 5.85 × 10^−04^	1.04 × 10^−03^ 7.35 × 10^−04^
Cd	Winter Summer	1.60 × 10^−03^ 1.30 × 10^−03^	7.70 × 10^−02^ 6.25 × 10^−02^	1.16 × 10^−01^ 9.37 × 10^−02^	1.44 × 10^−01^ 1.17 × 10^−01^	1.80 × 10^−03^	2.88 × 10^−06^ 2.34 × 10^−06^	1.39 × 10^−04^ 1.23 × 10^−04^	2.09 × 10^−04^ 1.69 × 10^−04^	2.59 × 10^−04^ 2.11 × 10^−04^
Cr	Winter Summer	1.36 × 10^−03^ 1.24 × 10^−03^	6.55 × 10^−02^ 5.97 × 10^−02^	9.82 × 10^−02^ 9.59 × 10^−02^	1.23 × 10^−01^ 1.12 × 10^−01^	8.40 × 10^−02^	1.14 × 10^−04^ 1.04 × 10^−04^	5.50 × 10^−03^ 5.02 × 10^−03^	8.25 × 10^−03^ 8.06 × 10^−03^	1.03 × 10^−02^ 9.41 × 10^−03^
Ni	Winter Summer	3.59 × 10^−03^ 3.08 × 10^−03^	1.72 × 10^−01^ 1.48 × 10^−01^	2.58 × 10^−01^ 2.22 × 10^−01^	3.23 × 10^−01^ 2.77 × 10^−01^	2.40 × 10^−04^	8.61 × 10^−07^ 7.39 × 10^−07^	4.13 × 10^−05^ 3.55 × 10^−05^	6.19 × 10^−05^ 5.33 × 10^−05^	7.75 × 10^−05^ 6.65 × 10^−05^
Pb	Winter Summer	4.84 × 10^−03^ 3.33 × 10^−03^	2.32 × 10^−01^ 1.60 × 10^−01^	4.65 × 10^−01^ 3.19 × 10^−01^	4.36 × 10^−01^ 3.00 × 10^−01^	1.20 × 10^−05^	5.80 × 10^−08^ 3.90 × 10^−08^	2.78 × 10^−06^ 1.92 × 10^−06^	2.16 × 10^−05^ 3.83 × 10^−06^	5.23 × 10^−06^ 3.60 × 10^−06^
	Winter Summer					Total ECLR	1.29 × 10^−04^ 1.15 × 10^−04^	6.24 × 10^−03^ 5.57 × 10^−03^	9.38 × 10^−03^ 8.87 × 10^−03^	1.17 × 10^−02^ 1.04 × 10^−02^

## Data Availability

Not applicable.
